# Personalized Cancer Care Conference

**DOI:** 10.3390/jpm3020070

**Published:** 2013-04-29

**Authors:** Kurt S. Zänker, Enrico Mihich, Hans-Peter Huber, Anne-Lise Borresen-Dale

**Affiliations:** 1 Department of Medicine, Faculty of Health Sciences, Institute of Immunology and Experimental Oncology, ZBAF, University Witten/Herdecke, Witten 58448, Germany; 2 Fritz-Bender-Foundation, Munich 80803, Germany; 3 Dana Faber Cancer Institute, Harvard University Medical School, Boston, MA 02115, USA; 4 Institute for Cancer Research, Norwegian Radium Hospital, Oslo University Hospital, Oslo N-0310, Norway

**Keywords:** personalized/individualized medicine, four-P-medicine, flood of information, genome-wide association studies, allele specific copy number analysis of tumors, single nucleotide polymorphism, kateagis, driver genes, passenger genes, cancer stem cells, supportive and psychological cancer care

## Abstract

The Oslo University Hospital (Norway), the K.G. Jebsen Centre for Breast Cancer Research (Norway), The Radiumhospital Foundation (Norway) and the Fritz-Bender-Foundation (Germany) designed under the conference chairmen (E. Mihich, K.S. Zänker, A.L. Borresen-Dale) and advisory committee (A. Borg, Z. Szallasi, O. Kallioniemi, H.P. Huber) a program at the cutting edge of “PERSONALIZED CANCER CARE: Risk prediction, early diagnosis, progression and therapy resistance.” The conference was held in Oslo from September 7 to 9, 2012 and the science-based presentations concerned six scientific areas: (1) Genetic profiling of patients, prediction of risk, late side effects; (2) Molecular profiling of tumors and metastases; (3) Tumor-host microenvironment interaction and metabolism; (4) Targeted therapy; (5) Translation and (6) Informed consent, ethical challenges and communication. Two satellite workshops on (i) Ion Ampliseq—a novel tool for large scale mutation detection; and (ii) Multiplex RNA ISH and tissue homogenate assays for cancer biomarker validation were additionally organized. The report concludes that individual risk prediction in carcinogenesis and/or metastatogenesis based on polygenic profiling may be useful for intervention strategies for health care and therapy planning in the future. To detect distinct and overlapping DNA sequence alterations in tumor samples and adjacent normal tissues, including point mutations, small insertions or deletions, copy number changes and chromosomal rearrangements will eventually make it possible to design personalized management plans for individualized patients. However, large individualized datasets need a new approach in bio-information technology to reduce this enormous data dimensionally to simply working hypotheses about health and disease for each individual.

## 1. Goals

The international faculty provided a superb opportunity for in-depth discussions within the arising field of personalized medicine in oncology. Leading experts in basic science and clinical oncology presented transdisciplinary strategies and concepts that might result in better, individualized outcomes and benefit for a cancer-diseased patient. The symposium focused on the up-coming and important issue of developing individualized treatment of cancer based on genetic and molecular profiles of individualized patients and their tumor.

## 2. The Meeting Focused on the Following Issues

(1): The management of a cancer patient will be driven in the future by an integral of individual tumor biology, tumor microenvironment, host characteristics as well as psycho-oncologic and cognitive behavior and social entities. 

(2): The management of a cancer patient will be driven in the future to identify that patient who will show a favorable therapeutic response, based on the individual biological dataset of the tumor, using high accuracy, reliable and robust prediction models.

(3): The management of a cancer patient should be individualized, evidence-based, and should provide the best care for cancer cure, metastasis prevention, symptom control and quality of life. Unfortunately, this goal has not been yet achieved for the majority of tumor types. Its feasibility has already been indicated by chronic myelocytic leukemia and cancers. Integrated and personalized medicine in oncology is no longer a dream, although there is still a gap between clinical interventions, which need to be taken into consideration, and what is happening in a patient’s day-to-day reality. Integrative and personalized medicine is one promising model to resolve the discrepancy between medical theory and clinical and individual achievements.

## 3. Novel Aspects of the Meeting

The discovery of driver and passenger genes, the description of about 1,400 aberrant molecular pathways involved in carcinogenesis, and the continuous detection of prognostic markers revolutionize the diagnosis, therapy and prevention of a cancer disease. However, there is an urgent need to discuss with and within all groups of a society—e.g., basic researcher, clinicians, health care providers and politicians—basic-science results, clinical trial modalities and outcome validation, biobanking, ethical and legal aspects for health and disease research.

(1): The meeting united different current views and attitudes in personalized cancer. The international and distinguished faculty members have explored interdisciplinarily the scientific results and the ethical and legal opinions of personalized cancer care.

(2): The results of the meeting summarized the aforementioned vignettes which are intended in the future to demonstrate the best individualized treatment plan and take into account patient preference and clinical acumen and the best multi-expertise available evidence.

## 4. Meeting Content

In his keynote lecture, **Leroy Hood**, (Seattle, USA) gave a talk entitled: “Systems Cancer Medicine: Towards Realization of Predictive, Preventive, Personalized and Participatory (P4) Medicine.” In the foreseeable future, clinicians, biomedical researchers, patients and consumers will be increasingly confronted with a flood of information, e.g. whole genome sequences, molecular profiling of diseased tissues, and multi-analytic blood testing of biomarker panels. The bio-convergence of these large datasets will enable prediction of disease susceptibility, early diagnosis to design actionable preventive schema, and personalized treatment regimens tailored to each individual [1]. He predicts that systems approaches will empower the transition from conventional reactive medical practice to a more proactive medicine. The vision for P4 medicine is that each individual/patient will be associated with a virtual data cloud of billions of data points and that the bio-information technology will be available to reduce this enormous data dimensionally to simple hypotheses about health and/or diseases for each individual. This reflects a new paradigm of a “holistic systems approach of the termed P4 medicine,” which might be complemented to a P5 medicine by introducing the term “prognosis” as suggested by the audience. The bottom message was: “Store the data in the clouds, share them with scientists worldwide and one will get a lot of answers for the progress of life science to move forward and look at nature’s unique concept of truth.”

### 4.1. Session 1: Genetic Profiling of Patients, Prediction of Risk, Late Side Effects

**Sir Bruce Ponder** (Cambridge, UK) gave the Thoresens Foundation lecture, “Clinical applications of genome-wide association study data: lessons from breast and prostate cancer.” To date, 22 common breast cancer susceptibility loci have been identified accounting for about 8% of the heritability of the disease. From two independent genome-wide association studies (GWAS) his group identified three new breast cancer risk loci at 12p11, 12q24 and 21q21 [2]. Rs10771399 (PTHLH) has a crucial role in mammary development and the establishment of bone metastasis, and rs2823093 (NRIP1) encodes an ER cofactor and has a role in the regulation of breast cancer cell growth. His group also identified a new functional prostate risk variant at the chromosome 8q24 upstream of the MYC oncogene. Rs378854 allele G reduces binding of the transcription factor YY1 and is associated with increased expression of the promotor PVT1 located 0.5 Mb downstream. Measurement of gene expression patterns in normal tissues from which cancer arises can be used by individuals and can be applied to stratification of populations, such as for screening. At present, the identified genetic variants can only explain about 14% (breast) or 30% (prostate) of the estimated polygenic variance.

**Vessela Kristensen** (Oslo, Norway) spoke about “Allele specific expression—Can it lead us to the risk allele?” She described a unique bioinformatics approach, ASCAT (allele-specific copy number analysis of tumors), to accurately dissect the allele-specific copy number of solid tumors, simultaneously estimating and adjusting for both tumor ploidy and non-aberrant cell admixture, a prerequisite to analyze correctly single nucleotide polymorphism (SNP) of cancer samples [3]. Using the ASCAT algorithm, her group studied the influence of germline and somatic variations on mRNA in ninety-two breast carcinomas. They detected 4,364 unique genes the expression of them was associated in cis with genetic variation and SNPs correlated with the expression of 8.8% of these genes. A novel finding was that 5.6% of the variation in expression was allele specific with respect to copy numbers.

**Eiliv Lund** (Tromso, Norway) presented preliminary data on the time dependent gene expression in peripheral blood from six years before diagnosis until time of diagnosis given at the first biopsy. The Norwegian Women and Cancer Study (NOWAC) post-genome biobank is one of a few larger prospective studies with blood and tissue samples suitable for whole genome transcriptomics. The data set of NOWAC supports the hypothesis that the gene expression in peripheral blood could serve as functional markers of the carcinogenic process and may no longer be wishful thinking.

**Patricia Ganz** (Los Angeles, USA) put forward the question why there is an increasing interest in the study of the late effects of cancer treatment. There are more than 28 millions cancer survivors worldwide. Beyond persistent symptoms, there are late toxicities in vital organs, which are strongly associated with the intensity of treatments. Therefore, there is an ongoing need for medical and psychological care for cancer survivors [4]. Cancer fatigue is the most common side effect of cancer and cancer treatment; cancer-related fatigue is only marginally investigated and is mainly caused, among others, by demographic factors, psychosocial factors and co-morbidity symptoms. She introduced the UCLA Mind-Body study and identified first predisposing risk factors for a severe cancer-related fatigue syndrome, which are identified as SNPs in the promotor regions of TNF-alpha, TNF-alpha receptor, IL-1 and IL-6.

### 4.2. Session 2: Molecular Profiling of Tumors and Metastases

**Michael Stratton** (Hinxton, UK) showed that all cancers carry somatic mutations and, at least, 415 genes are known to be causally involved in human oncogenesis [5]. He spoke about driver mutations, which convert a normal cell into a cancer cell and 1 to 10 mutations, or more, gives the cancer cell a clonal growth advantage, whereas passenger mutations, 100 to 1,000, or more, are a reflection of the number of mitoses. He introduced a catalog of somatic driver mutations from 21 breast cancers and applied mathematical methods to extract mutational signatures of the underlying processes. He showed that multiple distinct single- and double-nucleotide substitutions signatures were discernible. In addition, a remarkable phenomenon of localized hypermutation, termed “*kateagis*” was observed. Regions of *kateagis* differed between cancers but usually co-localized with somatic rearrangements. The mechanisms underlying most of these mutational signatures are unknown, but a role of apolipoprotein B mRNA editing enzyme, catalytic polypeptide-like (APOBEC) family of cytidine deaminases is proposed. 

**Jun Wang** (BGI-Shenzhen, China). He stated that cancer is a game of cell evolution and nothing in cancer biology makes sense except in the sense of evolution, which is a Darwinistic view. Hereby, he referred to one of the most famous essays by Theodosius Dobzhansky (1900–1975): “Nothing in Biology Makes Sense Except in the Light of Evolution.” (*The American Biology Teacher* 35: 125–129; 1973). Cancer cells are very personalized and this is a rationale for single cell genetic profiling [6]. He described a method for analyzing the cancer genome at a single cell nucleotide level, which allows the initial characterization of the disease-related genetic architecture at the single cell nucleotide level. He exemplified this approach by analyzing hepatocellular carcinomas, which frequently carry JAK1 mutations. Patients showing an early integration of hepatitis B virus into tumor liver cells have a shorter lifespan when there is a stroke by HCC. The big challenge for the future will be to come from “Omics” to personalized medicine.

**Lao Saal** (Lund, Sweden) introduced the South Sweden Cancerome Analysis Network-Breast Initiative (SCAN-B). To date, over 2,300 women have been enrolled in SCAN-B, with consistent patient accrual of 20 to 30 patients per week. In the first phase, genome-wide gene expression profiling by next-generation mRNA sequencing has commenced. This initiative should significantly reduce the time to discovery, validation, and clinical implementation of more powerful diagnostic, prognostic and predictive tests for breast cancer.

**Zoltan Szallasi** (Lyngby, Denmark). There is an urgent need for predictive biomarkers to determine the sensitivity to certain chemotherapy drugs, such as cisplatin. He demonstrated that cisplatin has a revival as a chemotherapy agent in patients with and without breast cancer type 1 susceptibility protein (BRCA1) gene mutations. Mutations in BRCA genes cause defects in DNA repair that predict sensitivity to DNA damaging agents, including platinum. The number of subchromosomal regions with allelic imbalance extending to the telomere (N(tAI) [7] predict cisplatin sensitivity *in vitro* and pathological responses to preoperative cisplatin treatment in patients with triple-negative breast cancer (TNBC). There is an ongoing trial in TNBC, taxane *versus* platinum-based therapy. Lysosome associated protein transmembrane 4 beta (LAPTM4b) is also a cancer-associated gene and the amplification of LAPTM4b was found to be a biomarker indicating a treatment resistance to doxorubicin and an increased risk of recurrence of breast cancer. LAPTM4b overexpression is also a novel independent prognostic marker for metastatic ovarian tumor and promotes autophagy and tolerance to metabolic stress in cancer cells.

**Carlos Caldas** (Cambridge, UK) brought some good news for breast cancer: All in all, the 10-year survival rate is 80%, but this success story is bought by an overtreatment of many women; however, within this context, tamoxifen saved more lives than any other therapy [8]. An integrated analysis of copy number variants and single nucleotide polymorphism and acquired somatic copy number aberrations (CNAs) of 2,000 frozen breast cancer samples revealed novel subgroups. These include a high risk, estrogen receptor positive 11q13/14 cis-acting subgroup and a favorable prognosis subgroup devoid of acquired somatic copy number aberrations (CNAs). About 15% of breast cancers are driven by a T-cell receptor deletion-mediated adaptive immune response in the “CAN-devoid” subgroup and a basal-specific chromosome 5 deletion-associated mitotic network. He also reported about an estrogen receptor low cohort who relapsed very aggressively after five years’ survival. The integrative view of the genome and transcriptome of a breast cancer population provide a novel molecular stratification, derived from the impact of somatic CNAs on the transcriptome.

### 4.3. Session 3: Tumor-Host Microenvironment Interaction and Metabolism

**Mina Bissell** (Berkeley, USA) spoke about a three-dimensional thinking to discover new targets for therapy. She introduced the mammary gland as an ideal model organism for studying tissue specificity and gene expression in mammals [9,10]. The mammary acinus is made up of a layer of polarized epithelial cells which produce milk and which are surrounded by myoepithelial contractile cells. The two-layered structure is surrounded by a basement membrane. It is evident that the microenvironment, the extracellular matrix and the tissue architecture play a crucial role in directing functional differentiation of organs, e.g., the mammary gland. In her laboratory, a 3D mouse mammary gland model was developed which enables to gain a better understanding of the organization, development and function of the acinus, and to identify key molecular, structural, and mechanical cues important for maintaining mammary function and architecture. The 3D-architecture is a message in itself; when destroyed, it might lead to carcinogenesis. The 3D-structure controls genetics, metabolism and e.g., integrin activity and intracellular pathways. The phenotype is dominant over the genotype. In this context her laboratory detected that molecular family members with sequence similarity 83 (FAM83A and FAM83B) are oncogenic and confer resistance to EGRR-TKIs. When the protein, encoded by the human gene FAM83A is overexpressed in breast cancer, the overexpression goes with a poor prognosis.

**Arnold Levine** (Princeton, USA) spoke about somatic cell reprogramming, cancer stem cell differentiation, cancer stem cells and the prognosis of breast and prostate cancer. Fibroblasts are transferred by four transcription factors—c-myc, Sox2, Klf-4 and Oct-4—into induced pluripotent stem cells (iPSC) [11]. However, the efficiency is low, only 1 of 1,000 cells becomes a stem cell. The efficiency and kinetics can be increased when a p53 knock-out mouse model is used. Almost 80% of fibroblasts can be reprogrammed within five days. The inactivation of p53 functions enhances the efficiency and decreases the latency of producing induced pluripotent stem cell (iPSC) *in vitro*, as well. Different p53 mutant alleles affect the reprogramming process; little or no p53 activity favors the entire process of somatic cell reprogramming. Reactivation of p53 at any time point during the reprogramming process interrupted the formation of iPSCs and induced newly formed stem cells, which lead to a fundamental question: What is a stem cell? Every stem cell has a different signature to be called a stem cell. Embryonic profiling and stem cell cluster with p53 mutations give in general a worst clinical outcome in cancer patients. In clinical terms, according to the Gleason score, a Swedish cohort of prostate cancer patients with watchful waiting was compared with a heavily treated cohort at Memorial Sloan Kettering Cancer Center (MSKCC, NY). The clinical outcome was almost similar. The patients were classified based on their mRNA microarray signature profiles. A subset of patients manifested tumor cells with stem-like signatures together with p53 and phosphatase and tensin homolog (PTEN) inactivation and a poor survival; a second group with intermediate survival outcome, characterized by the TMPRSS2-ERG fusion, and a third group with benign outcome. This classification was independent from the Gleason score.

**Elaine Mardis** (St. Louis, USA) spoke about variable clinical features of estrogen-receptor positive breast cancer with somatic alterations. She investigated tumor biopsies from patients pretreated in two studies with neoadjuvant aromatase inhibitor therapy [12]. Eighteen significantly mutated genes were identified, including five genes—RUNX1, CBFB, MYH9, MLL3 and SF3B1—previously linked to hematologial disorders. Mutant MAP3K1 was associated with luminal A status, low-grade histology and low proliferation rate. Mutant GATA3 correlated with suppression of proliferation upon aromatase inhibitor treatment. Distinct phenotypes in estrogen-receptor positive breast cancer are associated with specific patterns of somatic mutations, which intercept with multiple cellular pathways.

**Larry Norton** (New York, USA) addressed personalized medicine. If personalized medicine is an answer for new therapies and changing clinical enigmas, then: (i) what are novel questions to be asked, (ii) what is the grade of complexity; and (iii) how do we deal with this complexity? The pervasive albatross of metastasis necessitates improved prevention and treatment of metastasis formation. He offers a new theory of metastasis formation, termed “self-seeding” [13,14]. The “self-seeding” paradigm, well validated in mathematical, experimental and animal models, challenges the notion that cancer cells that leave a primary tumor, unidirectionally seed metastases in regional lymph nodes and/or distant sites. In contrast, there is mounting evidence that circulating tumor cells can move multidirectionally, seeding not only at distant sites but also in the tumor of origin. A network of paracrine tumor-secreted factors, e.g., cytokine (Il-6) and chemokine (IL-8), contribute to tumor progression as attractants at the primary site. The paracrine signals between the carcinoma, myeloid and endothelial cells drive this process. Cancer cells overexpressing CXCL1 and CXCL2 attract CD11b(+)Gr1(+) myeloid cells into the tumor and produce chemokines including S100A8/9 that enhance cancer cell survival. Chemotherapeutic agents kill cancer cells and these treatments trigger a parallel stromal reaction by releasing TNF-alpha by endothelial cells and pericytes. TNF-alpha increases CXCL1 and CXCL2 expression in cancer cells and amplifies the CXCL1/2 S100A8/9 loop, which causes chemoresistance. CXCR2 blockers break this cycle and augment the efficacy of chemotherapy against breast cancer and particularly against metastasis.

### 4.4. Session 4: Targeted Therapy

**Gordon Mills** (Houston, USA) spoke about the challenges that multiple signaling pathways must be addressed and which are required to be inhibited to achieve effective personalized therapy, e.g., for those patients, whose cancers exhibit “PI3Kness.” Personalized medicine is to target aberrations that drive tumor growth and survival by administrating the right drug combination for the right person at the right time. There are numerous challenges that need to be addressed like tumor heterogeneity and molecular evolution, costs and potential morbidity of biopsies, lack of effective drugs against genomic aberrations and technical limitations of molecular testing. He demonstrated that the PI3K pathway is critically important to cellular function, but inhibition of the pathway at the level of mTOR or AKT results in the activation of potent feedback loops resulting in activation of multiple cell surface tyrosine kinases, PI3K itself and in the case of mTOR inhibitors AKT. PIK3CA/PTEN genomic aberrations and high p-AKT levels are associated with rapamycin sensitivity *in vitro* [15]. Rapamycin-mediated Akt activation is greater in rapamycin sensitive cells, with a similar observation in patients with clinical responses on exploratory biomarker analysis. This observation may contribute that mTOR inhibitors appear to make some tumors grow more rapidly, an unexpected and disappointing consequence of targeted therapeutics.

**Rene Bernards** (Amsterdam, The Netherlands) gave the EACR lecture on functional genetics and optimizing the treatment of cancer. Tumors harboring so-called driver mutations frequently exhibit striking sensitivities to inhibition of these oncogenic driver pathways, a principle referred to as oncogene addiction. Understanding drug resistance mechanisms will help design more efficient combination treatment strategies that help to block resistance mechanisms before they become clinically manifested. Fresh-frozen tumor samples from 381 colorectal cancer patients were collected and mutations in KRAS (30.2%), BRAF (11.0%), and PIK3CA (11.5%) were assessed. The identified signature revealed mechanisms that can activate ERK/MAPK pathway in KRAS, BRAF and PIK3CA wild-type patients. The combined signature is associated with response to cetuximab treatment in patients with metastatic colorectal cancer [16]. A combined oncogenic pathway signature identifies patients with an active EGFR signaling pathway that could benefit from downstream pathway inhibition.

**Joe Gray** (Portland, USA) spoke about Omics and about system approaches to targeted therapy beyond genomics. The micro- and nano-environments interact with the genomics of tumors via tumor-associated fibroblasts, pericytes and endothelial cells. There is an urgent need for algorithms and validated decision trees to describe genetic targets in tumors, because money and patients are limited. About 1,400 pathways have been identified to be involved in cancer. Protein signaling networks play a role in cellular function, and their dysregulation is central to many diseases, including cancer. To shed light on signaling network topology in specific contexts, such as cancer, requires interrogation of multiple proteins through time and statistical approaches to make interferences regarding network structures. Bayesian network inference algorithms hold particular promise in that they can capture linear, non-linear, combinatorial, stochastic and other types of relationship among across multiple levels of biological organization. His group applied these methods to reverse-phase protein array time-course data from a breast cancer cell line (MDA-MB-468) to predict signaling links that could be independently validated using targeted inhibition. The proposed method offers a general approach to elucidate molecular networks specific to a biological context, including, but not limited to, human cancers.

### 4.5. Session 5: Translational Research

**Olli Kallioniemi** (Turku, Finland) spoke about systems medicine and implementing individualized medicine in the clinic. The question is: How do we achieve progress in medicine? There are many obstacles, e.g., off-label use of medication, to find new indications for already old and established drugs, regulatory, ethical and educational concerns and time and money. Furthermore, there are business, administrative and social mindsets in order to bridge the gap between scientists, physicians and patients. The translation of genomics in cancer research to clinical treatment decisions remains a significant challenge and it may take years to achieve the optimized goal. One example is in acute myeloid leukemia (AML) where the number of sporadic mutations is high and variable from patient to patient, and detecting driver mutations remains a challenge. Many driver mutations are either not approached by drugs or there are no approved drugs available. He introduced an individualized concept for systems medicine in AML to implement personalized medicine in the clinical setting. Sequential samples of AML patients at different stages of disease progression were biobanked, exome and RNA sequences; phosphoproteomics profiling of the samples were also performed. Furthermore, the response of the patients’ AML cells *ex vivo* to a cancer pharmacopeia-wide panel of 270 cancer drugs was evaluated. An integrated database was built up and correlated to genomic and cell signaling changes in AML with *ex vivo* drug response on a patient-to-patient basis. Finally, promising observations from these combinations of many levels of cancer omics were translated to select approved drugs for individualized therapy optimization in the clinic.

**Anne-Lise Borresen** (Oslo, Norway) presented data from several high-throughput molecular analyses of DNA, mRNA and miRNA proteins and metabolic profiles of breast tumors, and how they could be used separately and combined in subdividing the patients into subclasses with improved prognostic potential. Classification built on levels of genomic distortions using two newly developed algorithms to measure whole-arm gains and losses (WAAI), and complex rearrangements (CAAI) were shown to correlate to outcome, and the prognostic power of the CAAI index was validated in the METABRIC cohort of 2,000 cases [17]. From the collaboration with the Sanger Center deep sequencing of 21 whole breast cancer genomes showed that the mutational processes evolve across the lifespan of a tumor, with cancer-specific signatures of point mutations and chromosomal instability often emerging late but contributing extensive genetic variation. Subclonal diversification was prominent, and every tumor studied had a dominant subclonal lineage, representing more than 50% of tumor cells. She then demonstrated that specific genetic alterations identified from such studies can be analyzed using a combination of immunofluoresence and fluorescence *in situ* (FISH) techniques (“double immunoFISH”) to identify intra-tumor heterogeneity. From 45 HER2 positive patients enrolled in a neo-adjuvant trial (3–4 FEC100 followed by 4 docetaxel plus trastuzumab, 3qw), tumor samples before and after therapy were analyzed, and changes in heterogeneity during the course of the treatment was followed and evaluated for response. By double immunoFISH technique, both phenotypic (ER and HER2 protein) and genomic changes (copy number of HER2 gene and centromere 17) were assessed in the cells simultaneously on biopsies before and after treatment. The patients with partial response displayed a high grad of cell-to-cell diversity regarding HER2 copy number, nuclear shape and size and the expression of the membrane protein HER2. This was in contrast to the results from the complete responders who showed a reduced diversity and were more frequently ER negative. In the patients with partial response, a higher diversity was seen after treatment. These results show the power of using data from high-throughput analyses of bulk tumors to explore cell-to-cell variations observed within tumors and utilize this information for personalized treatment. 

**Laura van’t Veer** (San Francisco, USA) talked about biomarker indication, which increases the likelihood of therapeutic responses. A microarray-based 70-gene prognosis signature (RASTER study) has improved the selection of patients with node-negative breast cancer for adjuvant systemic treatment. The 70-gene prognosis signature identified from a cohort of 585 eligible patients 219 patients with good and 208 patients with poor prognosis. She showed that the use of the prognosis signature is feasible in Dutch community hospitals. Adjuvant systemic treatment was advised less often when the more restrictive Dutch Institute for Health Care Improvement - CBO guidelines were used compared with the finally given after use of the prognosis signature. For other guidelines assessed, less adjuvant chemotherapy would be given when the data based on prognosis signature alone are used, which might spare patients from adverse effects and confirms previous findings.

### 4.6. Session 6: Informed Consent, Ethical Challenges and Communication

**Stephan Friend** (Seattle, USA) addressed a learning process in respect to the reality of building cancer models [18]. The challenge for the future will be to translate—catalyzed by networking—genomics mega-data into effective models of disease and improved healthcare. He said that efforts to develop effective biomarkers and therapies will be inefficient at best until we better understand diseases as altered bionetworks and view diseases at an individual patient level. Furthermore, network-based drug discovery aims to harness this knowledge to investigate and to understand the impact of interventions, such as candidate drugs, on the molecular networks that define the physiological or pathophysiological states. Ultimately, the right drug should be administered to the right patient at the right time. Focusing on well-studied pathways, refining the definition of disease, and identifying disease subtypes, he demonstrated a more holistic approach to elucidating human diseases, with the potential to revolutionize treatment of these diseases.

**Nance Guilmartin** (Boston, USA) began with a question: “How *do* you listen past a patient’s fear to hear what they need to know, are afraid to ask or don’t know they don’t know? And how can you step into their shoes if you haven’t been where they stand?” She shared research insights into communication in today’s 24/7 world where 80%–90% of what we say can be misinterpreted—often without our knowing it. This is especially true when facing life-changing conversations with patients and families considering clinical trials. Communication, she explained, is more than words. The meaning behind our words is dramatically changed depending upon our unspoken attitude, assumptions, body language, and our energy—the literal “wavelength” and frame of mind that affect what we convey to others. She encouraged us to take a moment, to allow ourselves to be fully “present”—like the nurse in the story she read from her book, *The Power of Pause* [19]. And she introduced us to trust-building practices that can quickly help us listen past a patient’s words to understand the unspoken and to confirm whether what we thought we said was interpreted the way we intended. 

**Sissel Rogne** (Bergen, Norway) introduced the Norwegian biobanks and addressed the questions about the ownership of frozen samples of tumor specimens with special reference to those specimens, which are further processed for scientific and clinical use [20]. There are ethical, legal and societal questions arising; above all, who should earn the benefit when biobank and health registry data in research are linked, and commercialized and public healthcare governance also has access to such data? She introduced 10 health registries that do not demand informed consent. She also addressed that the Icelandic population is a good target population for evaluating human health and disease entities, because the families are closely related, a fact, which is also true for the Indian casts. 

## 5. Conclusion

**Kurt Zänker** (Witten, Germany) summarized the meeting. Personalized medicine in oncology is no longer a dream; there is room for hope. However, there is still an urgent need: (i) to identify the benefits and harms of personalized medicine in oncology, compared with existing, still more generalized anti-tumor therapeutic strategies; (ii) to discuss the complex ethical and legal issues, including the possibility of discrimination of high risk-individuals and patient’s autonomy in relation to genetic testing of minors; (iii) to show transparency and clear communication addressing the right to know and not to know about risk-based screening processes; (iv) to develop new bio-information tools to handle the individual patient’s dataset, thereby optimizing individual therapeutic strategies, and; (v) to assess personalized cost-effectiveness and acceptability by health policy-making and health care actors, considering the implication of incorporating genetic information in any intervention strategy, and, with emphasis to considering the newly arising role of cancer stem cells [21]. 

## 6. Future Meeting

Kurt Zänker proposed, with the approval of the Fritz-Bender-Foundation (H.P. Huber), the Stiftelsen Kristian Gerhard Jebsen (J.V. Johannessen) and the Institute for Clinical Medicine UiO/OUS (A.L. Borresen-Dale), to repeat the meeting in three years’ time and to show the progress within the different research fields of interest by inviting again this outstanding scientific faculty. Farewell and thanks to all having made this remarkable meeting possible.
